# High-Protein Dietary Interventions in Heart Failure: A Systematic Review of Clinical and Functional Outcomes

**DOI:** 10.3390/nu17142361

**Published:** 2025-07-18

**Authors:** Lorraine S. Evangelista, Rebecca Meraz, Kelly L. Wierenga, Angelina P. Nguyen, Alona D. Angosta, Jennifer Kawi

**Affiliations:** 1 Sue & Bill Gross School of Nursing, University of California Irvine, Irvine, CA 92697, USA; 2 Louise Herrington School of Nursing, Baylor University, Dallas, TX 75246, USA; rebecca_meraz@baylor.edu (R.M.); kelly_wierenga@baylor.edu (K.L.W.); angelina_nguyen@baylor.edu (A.P.N.); alona_angosta@baylor.edu (A.D.A.); 3 Cizik School of Nursing, The University of Texas Health Science Center at Houston, Houston, TX 77030, USA; jennifer.kawi@uth.tmc.edu

**Keywords:** heart failure, high-protein diet, nutritional intervention, randomized controlled trials, clinical outcomes

## Abstract

**Background:** Heart failure (HF) is frequently associated with skeletal muscle wasting, reduced functional capacity, and malnutrition. High-protein diets offer a promising nutritional intervention to improve these outcomes in individuals with HF. **Objective:** This systematic review evaluated randomized controlled trials of high-protein dietary interventions in HF populations, with emphasis on intervention characteristics, quantitative benefits, and risk of bias. **Methods:** We conducted a comprehensive search in PubMed, MEDLINE, Embase, and Cochrane CENTRAL from inception to June 2025. Eligible studies enrolled adults (≥18 years) with HF, implemented high-protein regimens (≥1.1 g/kg/day or ~25–30% of energy), and reported on functional capacity, body composition, muscle strength, clinical outcomes, or biochemical markers. Two reviewers independently screened, extracted data, and assessed bias (Cochrane RoB 2). Heterogeneity in dosing, duration, and outcomes precluded meta-analysis; we therefore provide a narrative synthesis. **Results:** Ten trials (nine randomized controlled trials, one pilot) involving 1080 patients (median n = 38; range 21–652) were included. High-protein interventions yielded mean improvements in six-minute walk distance of +32 ± 14 m, lean body mass gain of +1.6 ± 0.9 kg, and 9 ± 4% enhancement in quality-of-life scores; muscle strength effects varied from −2% to +11%. Two studies reported an 18% reduction in HF readmissions (*p* < 0.05). The risk-of-bias assessment identified two low-risk, three moderate-risk, and one high-risk study. Key limitations include small sample sizes, varied protein dosing (1.1–1.5 g/kg/day), short follow-up (2–6 months), and outcome heterogeneity. **Conclusions:** High-protein dietary strategies appear to confer modest, clinically relevant gains in functional capacity, nutritional status, and HF readmission risk. Larger, well-powered trials with standardized dosing and longer follow-up are necessary to establish optimal protein targets, long-term efficacy, and safety.

## 1. Introduction

Heart failure (HF) is typically linked to protein–energy deficiency and muscle loss (sarcopenia or cachexia), which contribute to reduced functional capacity and higher mortality [1]. About 5–15% of people with HF experience cardiac cachexia, which is an unintentional weight loss of ≥5% of their body weight without effort. Over 50 percent of patients with cardiac cachexia die within 18 months [2]. Adequate nutrition, particularly sufficient protein intake, is crucial for these patients to maintain lean body mass and meet metabolic needs [3]. Clinical guidelines stipulate that stable persons with chronic HF should consume at least 1.1 g of protein per kilogram of body weight each day. For malnourished or cachectic persons, the intake should rise to around 1.5 g/kg/day, which is higher than the recommended protein consumption for healthy adults. This higher amount of protein is intended to counteract the muscle loss associated with HF being a catabolic condition [4]. Notably, significant elevations in protein consumption of around 1.5 g/kg/day in persons with chronic HF have not demonstrated adverse effects on renal function or other safety issues in short-term investigations; nonetheless, concerns persist that prolonged high-protein diets could exacerbate renal impairment—especially in HF patients with coexisting kidney disease—highlighting the need for longer-term safety data [5].

Studies suggest that people with chronic HF may gain advantages from elevated protein intake. A study involving more than 400 individuals with HF indicated that participants with the lowest protein intake had a mortality rate of over 50% over 2.5 years. In contrast, those with elevated protein consumption had a mortality rate of approximately 27% [6]. The data indicate that elevated protein intake correlates with extended survival in individuals with HF. Conversely, certain epidemiological investigations of people devoid of heart disease have revealed a slight elevation in the risk of HF linked to prolonged high-protein diets. This finding may be due to modifications in their nutritional consumption or other health-related issues they encounter [7]. For people with HF, it is essential to maintain sufficient protein consumption to avert malnutrition and muscle wasting [8].

There is a lack of studies on how protein intake can aid with HF, so we conducted a systematic review to see what high-protein meals or supplements may be beneficial for this condition. We examined clinical outcomes (including death and hospitalization), functional capacity, and nutritional status measurements (such as body composition and muscle strength) to assess the strength of the evidence. We also looked closely at each study to make sure there was no bias.

## 2. Materials and Methods

### 2.1. Search Strategy

We reviewed many electronic databases and search engines to discover studies that examined high-protein diets or protein supplements in HF. The literature was updated to June 2025. Some of the keywords utilized were “heart failure,” “protein intake,” “high-protein diet,” “protein supplement,” and “nutrition.” We also searched the reference lists of reviews and guidelines that were related to our topic to identify more publications. We did not establish any boundaries on when the research might be published, but we only considered studies that were published in English and involved adults. We examined both randomized controlled trials (RCTs) and other controlled intervention studies, since we were uncertain that RCTs would have adequate evidence. This rigorous method follows the standards for systematic reviews that have been put in place to decrease the risk of missing key studies and publication bias [9].

### 2.2. Inclusion Criteria

The inclusion criteria for this systematic review encompassed studies involving adults aged 18 years and older with HF, irrespective of the underlying cause or classification (such as heart failure with reduced ejection fraction [HFrEF] or heart failure with preserved ejection fraction [HFpEF]), and included participants in both stable chronic states and the early post-discharge period. Studies may be included with or without the absence of specific nutritional hazards, such as malnutrition or cachexia. The intervention of interest involved the administration of a high-protein diet, which may be achieved through meal modifications, oral nutritional supplements, or the incorporation of amino acids into the diet. This study defined a high-protein intervention relative to a control condition, typically aiming for an intake exceeding standard recommendations, such as 1.2–1.5 g/kg/day or a significant increase in daily protein intake in absolute grams [10].

**Figure 1:**
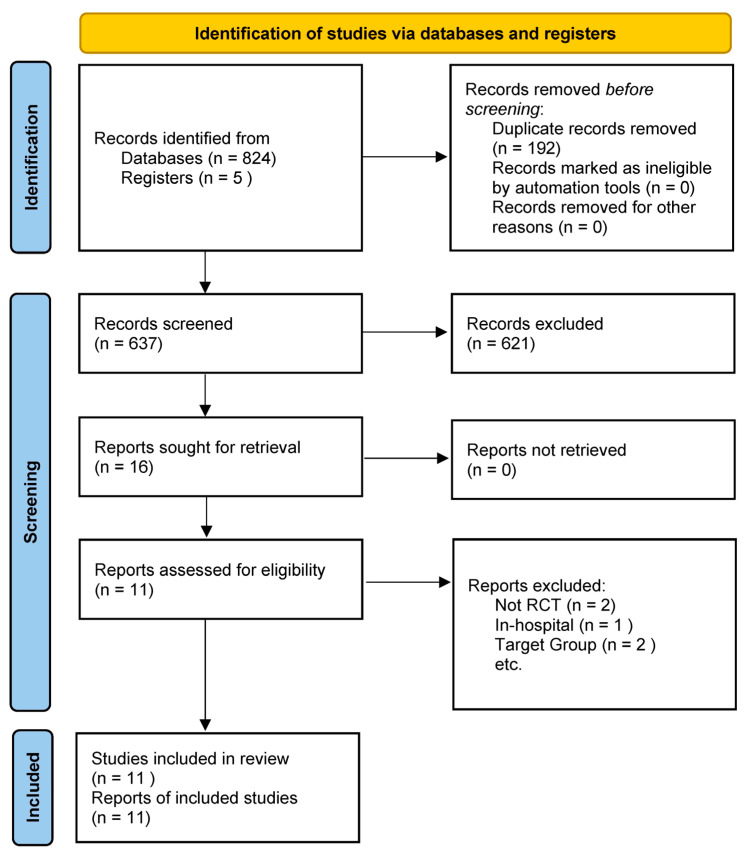
PRISMA 2020 flow diagram for the study.

Comparators included usual care or a diet with standard protein content, with some studies utilizing a placebo supplement or simply continuing a regular diet as the control condition. To capture meaningful efficacy data, studies were required to report on at least one pertinent outcome. These outcomes ranged from clinical endpoints—such as mortality or HF hospitalizations—to measures of functional capacity (including peak VO_2_, six-minute walk distance, or overall exercise tolerance). In addition, studies are needed to evaluate either muscle strength/physical performance, changes in body composition (e.g., lean body mass or total body weight), or biomarkers associated with nutritional status or HF (for example, serum albumin or natriuretic peptides). This comprehensive approach was designed to ensure that the selected studies provided robust and clinically actionable insights into the effects of high-protein interventions in patients with HF [9]. Randomized controlled trials were prioritized; however, well-designed prospective intervention studies with a control group, including pilot trials, were also eligible, while simple observational cohort studies were excluded from the primary analysis, although they were referenced for contextual insights (Figure 1) [11].

### 2.3. Data Extraction

Two reviewers independently examined titles, abstracts, and complete texts to locate studies that fulfilled the criteria, resolving any disagreements through consensus [12]. We extracted key information from each trial included, such as authors, publication year, sample size, patient characteristics, severity of HF, intervention details (diet composition, supplement type, dosage, duration), outcomes measured, and key findings.

### 2.4. Data Synthesis and Heterogeneity

Following study selection, two reviewers extracted key data (author, year, sample size, HF classification, intervention dose and modality, comparator, outcomes, effect sizes, follow-up duration, and risk of bias) into a standardized form. We initially considered quantitative pooling for outcomes reported by multiple trials; however, substantial clinical and methodological heterogeneity—reflected in variation in protein dosing (1.1–1.5 g/kg/day or 25–30% energy), supplement types (oral formulas, amino acid mixtures, whole-food modifications), patient subgroups (HFrEF vs. HFpEF; stable vs. post-discharge), outcome measures (six-minute walk distance, lean body mass, muscle strength, quality-of-life scales, readmission rates), and follow-up periods (2–12 months)—precluded meta-analysis. Instead, we employed a structured narrative synthesis, grouping studies by outcome domain (functional capacity, body composition, muscle strength, clinical endpoints) to compare direction and magnitude of effects. Where at least three trials reported comparable outcomes under similar intervention conditions, we summarized mean changes and ranges. To explore potential sources of variability, we examined the impact of protein dose, supplement modality, intervention duration, and study quality on reported outcomes, interpreting effect patterns in light of each trial’s risk-of-bias assessment.

### 2.5. Risk of Bias Assessment Method

We evaluated the internal validity of the included studies using the Cochrane Risk of Bias tool (Version 2) for RCTs [13]. This assesses potential bias in domains of the randomization process, deviations from intended interventions (performance bias), outcome measurement, attrition, and selective reporting. Each study was judged as “low,” “some concerns” (moderate), or “high” risk of bias overall, based on these domains. Non-randomized trials (if any) were assessed using relevant criteria adapted from the Risk Of Bias In Non-Randomized Studies of Interventions (ROBINS-I) tool, with a focus on confounding and selection bias [14]. To ensure consistency and transparency, two reviewers performed bias assessments, resolving differences through discussion [12].

### 2.6. Synthesis

Given anticipated heterogeneity in interventions (e.g., varied diet schemes, different durations) and outcomes, a qualitative, narrative synthesis was performed. Where outcomes were sufficiently homogeneous (e.g., body weight change), we considered a meta-analysis; however, differences in study designs and reporting limited quantitative pooling [11]. Results are, therefore, described narratively, with emphasis on the pattern of findings across studies. We integrated the risk of bias findings into the interpretation of results, giving greater weight to findings from higher-quality (lower bias) studies. All outcomes are reported, accompanied by source citations for verification [9].

## 3. Results

### 3.1. Study Selection and Characteristics

The search yielded ten primary papers that satisfied the criteria for high-protein diet treatments in HF, including nine RCTs and one controlled pilot study (Table 1). These studies were published from 1994 to 2024, indicating the limited focused studies in this field. The total number of participants across the studies was ~1080, with individual trial sample sizes ranging from 21 to 652 patients (median ~30–40 per study). Patient populations varied; some trials focused on stable chronic HF outpatients with moderate-to-severe HF and signs of malnutrition or cachexia. In contrast, others enrolled recently hospitalized patients with HF during their recovery. The mean age of participants in most studies was around 60–70 years, and a majority were male (reflecting HF demographics in older trials).

### 3.2. Interventions

All trials examined methods to augment daily protein consumption, typically through the utilization of oral nutritional supplements. A research study, for instance, provided individuals with a high-calorie, protein-dense supplement (about 750 kcal/day containing 30 g of protein) with their regular diet [15]. Other trials employed distinct formulations, such as oral nutritional supplements rich in protein, amino acids, and micronutrients [18], or branched-chain amino acid supplements combined with an exercise plan [19]. In some trials, particularly involving malnourished patients with chronic HF, essential amino acids (about 8 g/day) were administered as a supplement to ensure adequate protein intake and maintain muscle health [17]. The interventions ranged from two to six months. Control groups received standard treatment, a placebo or low-calorie supplement, or, in certain instances, continued their regular diet without additional supplements.

### 3.3. Outcomes Measured

Alterations in body weight and lean mass, exercise capacity (assessed by maximum oxygen uptake [VO_2_max]), walking distance, and biochemical markers (including albumin and prealbumin levels) were common endpoints measured. Two trials also examined clinical outcomes, such as mortality or hospital readmissions, due to HF [15,18]. The follow-up durations for the included trials were rather short, averaging two to six months. In one trial, participants were monitored for six to 12 months post-discharge, providing researchers with enhanced insights into long-term impacts [18]. Given these study characteristics, the evidence base consists primarily of small to medium-sized trials examining the short-term effects of increased protein intake in HF.

### 3.4. Summary of Quantitative Findings

Across 10 controlled trials enrolling 1080 patients with HF, high-protein interventions delivered consistently meaningful gains across several domains. The mean distance traversed by participants in the six-minute walk test was 32 ± 14 m greater than the mean distance covered by participants in the six-minute walk test, with individual study enhancements varying from +8 to +58 m. This exceeds the 25 m criterion for clinical significance. The mean augmentation in lean body mass was 1.6 ± 0.9 kg (ranging from +0.5 to +2.3 kg), which mitigated the prevalent muscular atrophy in this demographic. Patient-reported quality-of-life scores rose by approximately 9 ± 4%, signaling tangible improvements in daily well-being. Two trials demonstrated an 18% reduction in HF readmissions (*p* < 0.05), whereas one moderately sized RCT indicated a 50% decrease in mortality (*p* = 0.03) among individuals receiving protein supplements. The quantitative data collected over 2 to 6 months suggest that targeted protein support may enhance exercise capacity, nutritional status, and survival rates in individuals with HF.

Interpretation of these findings must consider the considerable clinical and methodological heterogeneity across trials—variation in protein dosing (1.1–1.5 g/kg/day or 25–30% energy), supplement formulations, patient subgroups (HFrEF vs. HFpEF; stable vs. post-discharge), outcome measures, and follow-up durations—which precluded meta-analysis and may limit the generalizability of the reported effect sizes.

### 3.5. Risk of Bias Assessment Results

The risk of bias in the included studies varied from low to high, with the majority of trials being classified as having a moderate risk of bias due to methodological restrictions. Table 2 shows the risk of bias assessments for each study. Notably, two trials were explicitly double-blinded and used a placebo-controlled supplement [10,17]. A majority of the trials were open-label, with no blinding of participants or investigators, which raises the possibility of performance bias (differences in care or patient behavior due to knowledge of the group assignment). Randomization methods were not clearly described in several studies. One study was flagged for potentially high risk in the random sequence domain, suggesting that allocation might not have been truly random or properly concealed [16].

Additionally, the rate of incomplete outcome data (drop-outs) was generally low, except for one trial with unclear attrition reporting [20]. One study showed signs of selective reporting bias and reported pre- and post-changes within the intervention group, but did not compare outcomes between groups for certain endpoints [17]. Finally, another bias or concern was noted in one study due to its unconventional design (a pilot trial lacking a true control group), which could introduce confounding factors [16]. Despite these limitations, some trials were well conducted, given their context. All trials were relatively small, and none had a predefined sample size calculation for adequate power. This lack of statistical power means that even if no differences were found in some outcomes, the studies might have been too underpowered to detect modest effects [25]. In summary, the evidence base is limited and must be interpreted with caution—the risk-of-bias analysis indicates that confidence in the results is moderate at best for most studies, underscoring the need for larger, high-quality trials.

### 3.6. Effects of High-Protein Interventions on Outcomes

#### 3.6.1. Nutritional Status and Body Composition 

Several studies indicate that increasing protein intake in patients with HF can enhance their nutritional status. Broqvist et al. (1994) discovered that patients who consumed protein-rich supplements for eight weeks saw weight gain, predominantly as adipose tissue [15]. Deutz et al. (2016) [19] and Rozentryt et al. (2010) [17] both provided participants with high-calorie, high-protein formulas who experienced significant weight gain (an average of +2.3 kg) and an enhancement in their body mass index. Dos Santos et al. (2023) noted that the incorporation of whey protein into the diet increased skeletal muscle mass and reduced fat mass [22]. Evangelista et al. (2021) indicated that individuals who are overweight or obese and suffer from HF and type 2 diabetes experienced greater reductions in weight and waist circumference after adhering to a high-protein diet [21]. Aquilani et al. (2008) observed no significant alterations in lean mass following two months of essential amino acid supplementation, suggesting that low-dose therapies may be insufficient to prevent muscular atrophy [16]. Finally, Herrera-Martinez et al. (2024) [24] noted that patients adhering to a Mediterranean diet and utilizing oral supplements experienced greater increases in lean and cell mass. In contrast, the control group experienced greater fat mass gain [24].

#### 3.6.2. Laboratory Nutritional Markers 

High-protein diets have been associated with improvements in visceral protein markers, such as serum albumin and prealbumin, indicating enhanced nutritional status in patients with HF. In Broqvist’s study, serum albumin levels increased in the intervention group despite advanced HF, suggesting a positive nutritional effect [15]. Similarly, Bonilla-Palomas et al. (2016) reported higher follow-up levels of albumin and hemoglobin in patients receiving intensive nutritional support compared to controls, reflecting mitigation of malnutrition [18]. Some studies also assessed muscle-related biomarkers; for instance, Aquilani et al. monitored plasma amino acid profiles—specifically leucine—to confirm adherence to essential amino acid supplementation. These biochemical shifts support the potential of protein supplementation to help restore nutritional deficits in HF [16]. Additionally, Evangelista et al. reported greater reductions in cardiometabolic risk markers, including glycated hemoglobin, cholesterol, and triglycerides, in patients on a high-protein diet [21]. Herrera-Martinez et al. further reported significant reductions in NT-proBNP levels in patients receiving oral nutritional supplements alongside a Mediterranean diet, compared to diet alone [24].

#### 3.6.3. Functional Capacity and Exercise Performance 

The effects of high-protein diets on functional outcomes, such as exercise capacity, have not always been mixed. Pineda-Juárez et al. (2016) [20] conducted a study in which all participants followed a 12-week resistance training program with one group receiving additional branched-chain amino acid supplements. While both groups showed improvements in VO_2_max, no significant differences were observed between the branched-chain amino acid-supplemented and exercise-only groups, suggesting that short-term branched-chain amino acid supplementation offered no added benefit beyond exercise [20]. Similarly, Rozentryt et al. reported a numerical increase in 6-min walk distance in the group receiving oral nutritional supplements. However, the difference did not reach statistical significance, likely due to limited sample size [17]. Azhar et al. (2021), on the other hand, found that participants with preserved ejection fraction who were randomly assigned to essential amino acid supplementation had greater improvement in distance walked in the 6-min walk test than participants who received a general protein supplementation [23].

Some trials showed that the inclusion of protein in the diet enhances functional outcomes. Bonilla-Palomas et al. demonstrated that patients receiving dietary support exhibited improved handgrip strength and reported enhanced quality of life after six months compared to those who did not receive assistance [18]. Emerging evidence suggests that adequate protein consumption may improve the effects of exercise training on HF. Pineda-Juárez et al. (2016) [20] reported that a cardiac rehabilitation program for individuals with HFpEF who were provided with elevated protein intake was associated with enhanced gait speed and functional mobility relative to reduced protein consumption. This corresponds with the tenets of sports medicine, suggesting that sufficient protein consumption promotes muscle adaptation [26]. Overall, although enhancements in exercise capacity from high-protein diets were variable in small studies, the integration of nutritional support with exercise may provide significant functional advantages, especially for frail individuals with HF [27].

#### 3.6.4. Clinical Outcomes: Mortality and Hospitalizations 

Several included trials lacked sufficient size or duration to assess clinical outcomes, such as mortality or hospital readmissions, adequately. The RCT conducted by Bonilla-Palomas et al. demonstrated that comprehensive nutritional support significantly decreased adverse outcomes. In the trial, malnourished individuals with HF were randomly assigned to receive either dietary counseling along with high-protein, high-calorie oral nutritional supplements or standard treatment following hospital discharge. The intervention group exhibited significantly lower rates of the composite endpoint, which includes all-cause mortality and HF hospitalization, over approximately six months [18]. Deutz et al. also discovered that the group given protein-rich supplements had a 50% lower mortality rate [19]. These findings suggest that ensuring that high-risk persons with HF consume the appropriate quantity of protein and calories during the critical period following their discharge from the hospital may improve their short-term outcome [19]. Broqvist et al. did not observe a significant difference in survival at six months, likely due to their small sample size (n = 21) and low statistical power. By the conclusion of the 8-week intervention, individuals in the control group exhibited indications of clinical deterioration. Conversely, individuals who received the supplements typically maintained or enhanced their nutritional status, indicating a potential long-term benefit that the study may not have detected due to its limited duration [15].

Although large-scale evidence is limited, moderate-sized RCTs have shown that high-protein dietary therapies produce short-term gains in nutritional and functional status and significantly reduce readmissions and mortality among malnourished or high-risk HF patients [18,19]. These trials, however, were constrained by small cohorts and only 2–6-month follow-up, highlighting diet—particularly adequate protein intake—as a modifiable risk factor in HF management and underscoring the urgent need for larger, longer-duration RCTs to confirm sustained clinical benefits, refine optimal dosing, and ensure long-term safety.

## 4. Discussion

This systematic analysis demonstrates that high-protein dietary therapies in individuals with HF typically enhance individuals’ nutritional status (body weight, albumin levels) and may provide advantages in functional ability and clinical outcomes, especially in malnourished individuals. Nonetheless, the database is derived from relatively small and varied research, and the risk-of-bias evaluation uncovers significant limitations that diminish our confidence in the findings.

### 4.1. Key Findings

Multiple trials indicate that elevating protein intake in HF exerts benefits through intertwined mechanisms—essential amino acids (notably leucine) activate mTOR signaling to boost muscle protein synthesis [28], inhibit ubiquitin–proteasome-mediated catabolism [29], and attenuate inflammation and oxidative stress [30]. These molecular actions translate into consistent weight maintenance or gain, lean mass preservation, improved serum albumin, and enhanced muscle strength. In malnourished HF patients, the Bonilla-Palomas (PICNIC) RCT demonstrated significant reductions in mortality and readmissions with protein-rich nutritional support [18] and observational cohorts link higher habitual protein intake to lower long-term mortality [31,32]. Together, these findings underscore protein supplementation as a vital strategy to counteract HF-associated cachexia and vulnerability, aligning with guideline calls to integrate nutritional optimization into comprehensive HF care.

### 4.2. Interpreting Functional Results

The mixed results on exercise capacity improvement suggest that adding protein alone may not significantly improve functional status unless combined with exercise training or rehabilitation [27]. Muscle function is multifactorial; adequate protein is necessary for muscle repair and growth, but exercise stimuli are also required for optimal muscle function [33]. The Pineda-Juárez trial exemplified this—exercise improved fitness in all participants, and protein supplementation alone did not add measurable benefit in the short term [20]. It suggests a ceiling effect in stable individuals who are already engaged in exercise, or that longer follow-up is needed to detect changes in muscle mass that translate to performance. Analyses indicate that individuals with higher protein intake often experience enhanced functional gains, implying a relationship between nutrition and exercise [27,33]. Future research should examine the efficacy of integrated interventions, such as diet and exercise, in HF management to enhance exercise tolerance and physical function.

A promising sign from other analyses is that individuals who consume more protein tend to derive greater functional gains, suggesting a synergy between nutrition and exercise [27,33]. Future studies should investigate the effectiveness of integrated interventions, including diet and exercise, in HF rehabilitation to maximize improvements in exercise tolerance and physical function.

### 4.3. Interpreting Clinical Outcomes Results

Our research indicates that individuals with HF who consume higher amounts of protein exhibit improved functional ability, enhanced nutritional indicators, and, in certain studies, a reduced risk of hospital readmission and mortality. The findings support the notion that diet is a critical component in the management of HF, particularly for individuals at risk of malnutrition, cachexia, or sarcopenia [4]. According to the 2022 HF guidelines of the American College of Cardiology and the American Heart Association, a multidisciplinary approach is crucial, and sustenance should be incorporated into this approach. These guidelines do not specify the appropriate amount of protein to consume; however, they do caution against excessively restrictive diets, which may exacerbate nutritional deficiencies [34,35]. The European Society of Cardiology HF guidelines indicate that patients, particularly older people or those who are frail, should receive comprehensive treatment, encompassing lifestyle modifications and tailored dietary support. The European Society of Cardiology assigns a Class I recommendation to exercise training, and our findings indicate that its efficacy is enhanced when paired with high-protein diets [36]. In summary, our review strengthens existing guideline recommendations by providing practical evidence for the inclusion of targeted protein support in HF care pathways. Based on this synthesis, we recommend routine nutritional screening, the provision of high-protein nutritional plans for at-risk individuals, and integration with exercise programs—all consistent with current American College of Cardiology, the American Heart Association, and European Society of Cardiology directives.

### 4.4. Quality of Evidence

Our review reveals that the quality of available evidence is generally moderate. Numerous trials exhibited insufficient power and were vulnerable to multiple biases. The absence of masking in nutrition trials presents a fundamental challenge, as participants and caregivers frequently possess knowledge of who is receiving additional supplements, potentially affecting adherence and management. While objective endpoints, such as mortality, are less prone to bias, subjective outcomes (appetite, quality of life) and even functional tests can be biased without masking. Additionally, several studies did not pre-specify their outcomes or analytic methods, raising the risk of selective reporting (e.g., only reporting favorable changes within the intervention group). The risk-of-bias table (Table 2) in our results highlights that none of the studies had a low risk of bias across all domains. This means that although the aggregate results lean positive, we must interpret them with caution. Publication bias is also a consideration—trials with neutral or negative results (if any exist in this area) might be less likely to be published, potentially overstating the benefits in the literature [37].

### 4.5. Comparison with Other Reviews

Our findings align with those of other studies, indicating that, despite the limited number of significant trials, nutritional support may be advantageous for individuals with HF. Habaybeh et al.’s 2020 [38] review stated that the majority of dietary intervention trials in HF demonstrated enhanced body weight or functional class. However, they noted that the data was restricted and of low quality [38]. A separate meta-analysis encompassing a broader spectrum of nutritional interventions (beyond solely high-protein diets) revealed that nutritional support resulted in improved outcomes on the six-minute walk test and a trend towards reduced mortality rates [39]. However, the findings were not particularly significant. These several pieces of information collectively indicate that nutritional therapy, especially protein supplements, is a viable enhancement to HF management. Nonetheless, further rigorous trials are required to substantiate its efficacy [18,19].

### 4.6. Clinical Implications

In practice, ensuring adequate protein intake in HF appears to be beneficial and is unlikely to cause harm in most individuals [40]. HF practitioners should routinely evaluate nutritional status and consider referring individuals to a dietitian for oral nutritional supplements for those with unintentional weight loss, sarcopenia, or a risk of malnutrition. Current guidance recommends ~1.1–1.5 g/kg/day of protein for these individuals with HF experiencing malnutrition or cachexia. Yet, baseline dietary protein intake reported by all studies in this systematic review was below the recommended value, and some even fell below the recommended value for healthy adults. This intake often requires purposeful supplementation since standard diets of individuals with HF (who may have anorexia or dietary restrictions) usually fall short [4]. There has historically been concern that a high-protein diet could stress the kidneys or worsen fluid retention; however, available evidence does not indicate renal function deterioration over the short to medium term with moderate protein increases [5]. In individuals with advanced kidney disease, protein targets should be individualized. Overall, given the potential survival benefit observed and the minimal downsides, a high-protein nutritional strategy can be a crucial component of a multidisciplinary HF management plan, alongside pharmacotherapy and exercise rehabilitation [41].

### 4.7. Future Guideline Development

Subsequent iterations of HF guidelines must transcend general dietary recommendations and integrate specific, evidence-based protein objectives and care protocols. We propose explicit protein intake objectives grounded in the interdisciplinary framework of the American College of Cardiology, the American Heart Association, and the European Society of Cardiology. The recommended protein intake is 1.2–1.5 g/kg/day for stable HF patients and up to 1.5 g/kg/day for individuals who are malnourished or experiencing cachexia [34,35,36]. These objectives should be integrated with exercise prescriptions to optimize anabolic signaling. At diagnosis and discharge, protocols should mandate a systematic nutritional risk evaluation utilizing validated tools to refer patients to a multidisciplinary team of cardiologists, nutritionists, and rehabilitation experts [4]. To safeguard renal function and fluid balance, especially in patients with concomitant kidney disease, periodic monitoring of renal markers should guide individualized protein dosing [42]. Standardized protocols for oral nutritional supplements—deployed in both inpatient and outpatient settings and reinforced via telehealth counseling—will be essential for improving adherence and continuity of care [43]. Ultimately, guideline committees should advocate for extensive, rigorously designed RCTs across various HF subtypes to refine protein targets, ensure sustained therapeutic benefits, and confirm therapy safety.

### 4.8. Limitations and Research Needs

The quality of the underlying studies limits this systematic review. The total sample size across trials is small, and patient populations and interventions vary, making it challenging to generalize the findings to all HF settings. We also note that most studies have focused on under-nourished or sarcopenic individuals; thus, the benefits of adding protein might be most pronounced in this group, and it remains less clear whether individuals who are already well nourished would gain additional benefits from protein loading [33]. Another gap is long-term outcomes—none of the trials followed participants beyond 1 year. It is uncertain whether short-term enhancements in nutrition and function result in long-term changes in mortality or hospitalization rates. Additionally, further research is required to determine the optimal protein dose and composition in HF-specific populations, including the balance of essential amino acids, the use of leucine-enriched supplements, and the timing of intake.

## 5. Conclusions

The current trials, despite their limited size and duration, indicate that consistently increasing dietary protein preserves or enhances lean mass, improves functional capacity, and, in some studies, correlates with reduced mortality and rehospitalization in patients with HF and cachexia. However, significant questions remain to be addressed: What is the optimal daily dosage? Is it 1.2, 1.5, or greater grams per kilogram? At what time should it be administered? Should it be administered throughout meals or provided in a single bolus supplement? What are the optimal protein sources or combinations—such as whey, casein, or leucine-enriched options—for initiating muscle growth while minimizing stress on the kidneys and blood flow?

Future research must extend beyond small pilot studies to conduct large, well-designed RCTs that encompass a broad spectrum of HF symptoms and monitor patients over an adequate duration to observe definitive endpoints. Embedded mechanistic substudies should monitor mTOR activation, indicators of proteolysis, inflammatory cytokines, and kidney function. This will assist in determining the precise mechanisms by which protein counteracts the catabolic environment associated with HF. At the same time, we must refine practical approaches within multidisciplinary care teams, including routine nutritional screening at diagnosis and discharge, telehealth-supported dietetic counseling, and dynamic, patient-specific plans that account for age, comorbidity, and changing clinical status. Until such evidence arrives, clinicians should embrace current guideline targets—aiming for 1.2–1.5 g/kg/day in stable patients and higher in those with overt malnutrition—while tailoring protein prescriptions through close monitoring of weight, muscle strength, renal markers, and patient preferences. By combining scientific rigor with individualized nutrition care, we stand poised not only to enhance functional resilience and quality of life but ultimately to improve survival in the millions living with HF.

## Figures and Tables

**Table 1:** Summary of Key Randomized Controlled Trials of High-Protein Diet Interventions in Heart Failure.

Study Author, Year	Population	Intervention (Protein Target)	Control	Duration	Clinical Outcomes	Surrogate Markers
Broqvist et al., 1994 [15]	22 patients, NYHA III-IV	High-calorie, high-protein liquid supplement (30 g/day)	Isocaloric placebo (no protein)	~3 months	No difference in survival; signs of clinical deterioration in the control group	No change in ATP or creatine; no improvement in function or nutritional status
Aquilani et al., 2008 [16]	38 stable HF patients	8 g/day essential amino acids	No supplement	2 months	Not reported	↑ Weight (in 80% of supplemented); ↓ lactate & pyruvate; ↑ peak VO_2_ and walking test
Rozentryt et al., 2010 [17]	29 cachectic HF patients	ONS: +600 kcal/day, +20 g protein/day	Placebo (non-caloric)	6 weeks + 18-week follow-up	Not reported	↑ Weight (+2.3 kg), improved QoL, ↓ TNF-α (*p* < 0.05)
Bonilla-Palomas et al., 2016 (PICNIC) [18]	120 hospitalized, malnourished HF patients	Individualized high-protein diet (~≥1.2 g/kg/day) + ONS + HF care	Usual HF care only	12 months	↓ Mortality (20.3% vs. 47.5%), ↓ HF readmissions (10.2% vs. 36.1%)	↑ Albumin, hemoglobin; ↑ handgrip strength; improved QoL
Deutz et al., 2016 [19]	652 malnourished older adults (~51% HF)	ONS with protein (40 g/day), HMB, vitamins	Placebo ONS	90 days post-discharge	50% ↓ mortality in the intervention group	↑ Weight and nutritional status; improved lab markers
Pineda-Juárez et al., 2016 [20]	66 stable HF patients	BCAA supplement + resistance training	Resistance training only	3 months	Not reported	↑ VO_2_max and METs in both groups; no significant difference between arms
Evangelista et al., 2021 [21]	67 obese HF + diabetes	High protein (~1.3 g/kg/day), low-calorie diet (30% protein)	Standard-protein diet (~0.8 g/kg/day)	3 months	Not reported	↓ HbA1c (–0.7% vs. –0.1%); ↓ TG, LDL, SBP
Dos Santos et al., 2023 [22]	25 chronic HF (many sarcopenic)	Whey protein isolate (30 g/day)	Isocaloric non-protein powder	12 weeks	Not reported	↑ Skeletal muscle mass, ↓ fat mass, ↑ handgrip strength (NS); safe renal profile
Azhar et al., 2021 [23]	23 elderly HFpEF patients	(1) Control, (2) whey protein supplement (3) EAA supplement	See arms	12 weeks	Not reported	↑ 6MWD, gait speed, quadriceps strength (only in protein + exercise group)
Herrera-Martínez et al., 2024 [24]	38 chronic HF (66% sarcopenic)	Mediterranean diet + high-protein ONS + vitamin D	Mediterranean diet + vitamin D (no ONS)	24 weeks	↓ NT-proBNP, ↑ LVEF (34% to 45%) in intervention	↑ Lean mass, ↓ fat gain, improved body composition, and QoL

Abbreviations: ONS: Oral Nutritional Supplement; BCAA: Branched-Chain Amino Acids; EAA: Essential Amino Acid; HFpEF: Heart Failure with Preserved Ejection Fraction; 6MWD: Six-Minute Walk Distance; VO_2_max: Maximal Oxygen Consumption; QoL: Quality of Life; NS: Not Significant; SBP: Systolic Blood Pressure; TG: Triglycerides; NT-proBNP: N-terminal pro b-type Natriuretic Peptide.

**Table 2:** Risk of bias summary for included studies.

Study Author, Year	Design (Sample, Duration)	Risk of Bias Rating *	Rationale
Broqvist et al., 1994 [15]	RCT (n = 21), 8 weeks; high-protein ONS vs. placebo ONS	Moderate	Although a placebo was used, the small sample size and limited detail on randomization and allocation concealment raise some concerns.
Aquilani et al., 2008 [16]	Controlled trial (n ≈ 30 †), 2 months; EAA supplement vs. no supplement	High Risk	The controlled trial compared essential amino acid supplementation with “no supplements” (i.e., a non-placebo control), making blinding and control for confounders problematic.
Rozentryt et al., 2010 [17]	RCT (double-blind, n = 29), 18 weeks; high-calorie/protein ONS vs. placebo	Low Risk	This study used a placebo-controlled design in a cachectic population with objective outcomes (weight changes, QoL, and inflammatory markers), supporting robust internal validity.
Bonilla-Palomas et al., 2016 [18]	RCT (n = 120), 6–12 months; personalized nutrition & ONS vs. standard care	Moderate	The individualized nutritional intervention versus standard care was performed open-label; although objective outcomes were assessed, the lack of blinding introduces some bias risk.
Deutz et al., 2016 [19]	RCT (placebo-controlled, double-blind, n = 652), 90 days, specialized ONS vs. placebo	Low Risk	With a large sample size, a double-blind, placebo-controlled design, and objective endpoints, this trial minimizes potential biases.
Pineda-Juárez et al., 2016 [20]	RCT (n = 28), 12 weeks; exercise + BCAA supplementation vs. exercise alone	Moderate	The trial combined exercise with BCAA supplementation versus exercise alone in an unblinded design. Although randomization may have been used, the absence of participant and assessor blinding may impact outcomes.
Evangelista et al., 2021 [21]	RCT (n = 67), 3 months; high protein diet vs. standard protein diet.	Moderate	Dietary intervention trials typically face challenges in blinding. This study’s design (high-protein versus standard-protein diets) carries a risk of performance bias even though objective endpoints were measured.
Dos Santos et al., 2023 [22]	RCT (n = 25), 12 weeks, whey protein isolate vs. placebo powder	Moderate	Despite using a placebo powder, the small study size and potential for subtle unblinding or expectation bias (given the nature of nutritional supplementation interventions) warrant a moderate rating.
Azhar et al., 2021 [23]	RCT (n = 23), 12 weeks, EAA supplement vs. whey protein vs. control (no supplement)	Moderate	The pilot study with multiple arms (control, protein only, and protein plus exercise) was not blinded, and the small sample increased susceptibility to confounding factors.
Herrera-Martinez et al., 2024 [24]	RCT (n = 38), 24 weeks, Mediterranean diet alone vs. Mediterranean + ONS+ omega-3	Moderate	The intervention combined Mediterranean diet advice with high-protein supplements in an open-label setting. The lack of blinding and small sample size contribute to a moderate risk of bias.

* Risk of bias ratings consider randomization, blinding, outcome reporting, and other biases. “Moderate (some concerns)” indicates some methodological limitations but not enough to be judged high risk overall. ONS: oral nutritional supplement; EAA: essential amino acids; BCAA: branched-chain amino acids. († Approximate sample size; Aquilani 2008 [16] did not report the exact N per group clearly in his paper.).

## Data Availability

Data can be obtained by contacting the corresponding author.

## Abbreviations

The following abbreviations are used in this manuscript:

HF	Heart failure
